# Uncertainty-driven dynamics for active learning of interatomic potentials

**DOI:** 10.1038/s43588-023-00406-5

**Published:** 2023-03-06

**Authors:** Maksim Kulichenko, Kipton Barros, Nicholas Lubbers, Ying Wai Li, Richard Messerly, Sergei Tretiak, Justin S. Smith, Benjamin Nebgen

**Affiliations:** 1 Theoretical Division, Los Alamos National Laboratory, Los Alamos, NM USA; 2 Center for Nonlinear Studies, Los Alamos National Laboratory, Los Alamos, NM USA; 3 Computer, Computational, and Statistical Sciences Division, Los Alamos National Laboratory, Los Alamos, NM USA; 4 Center for Integrated Nanotechnologies, Los Alamos National Laboratory, Los Alamos, NM USA; 5 Nvidia Corporation, Santa Clara, CA USA

**Keywords:** Atomistic models, Molecular dynamics, Chemical physics, Computational chemistry

## Abstract

Machine learning (ML) models, if trained to data sets of high-fidelity quantum simulations, produce accurate and efficient interatomic potentials. Active learning (AL) is a powerful tool to iteratively generate diverse data sets. In this approach, the ML model provides an uncertainty estimate along with its prediction for each new atomic configuration. If the uncertainty estimate passes a certain threshold, then the configuration is included in the data set. Here we develop a strategy to more rapidly discover configurations that meaningfully augment the training data set. The approach, uncertainty-driven dynamics for active learning (UDD-AL), modifies the potential energy surface used in molecular dynamics simulations to favor regions of configuration space for which there is large model uncertainty. The performance of UDD-AL is demonstrated for two AL tasks: sampling the conformational space of glycine and sampling the promotion of proton transfer in acetylacetone. The method is shown to efficiently explore the chemically relevant configuration space, which may be inaccessible using regular dynamical sampling at target temperature conditions.

## Abstract (web summary)

A biasing energy derived from the uncertainty of a neural network ensemble modifies the potential energy surface in molecular dynamics simulations to rapidly discover under-represented structural regions that meaningfully augment the training data set.

## Main

Machine learning (ML) is a firmly established approach in chemical science that demonstrates great promise for the acceleration of physical simulations. A particular strength of ML models is a robust representation of the potential energy surfaces of molecular and materials systems when trained to large and diverse data sets of high-fidelity quantum chemistry simulations. For example, ML-based potentials^1–11^ approach ab initio^12,13^ or density functional theory (DFT)^14^ levels of accuracy at a computational cost near that of classical force fields^15–17^. In recent years, various ML models, such as neural networks (NNs)^18–25^, Gaussian approximation potentials^26^, spectral neighbor analysis potentials^27^, moment tensor potentials^28^ and symmetric gradient domain ML^29,30^, have demonstrated remarkable success in the field of atomic-scale discovery.

No matter how sophisticated the ML model architecture, however, the quality and diversity of the training data remain crucial for ultimate model accuracy. Therefore, training sets for ML potentials need to span as much phase (structural) space as possible to perform meaningful simulations. Additionally, the training set needs to be as diverse as possible to avoid overfitting towards excessively represented training data (such as near-equilibrium configurations in MD trajectories).

Entropy-maximization techniques^31,32^ help to partially overcome these problems by maximizing the structural diversity of a data set. When acquiring new data, these methods are focused on the structural dissimilarity compared to existing data. However, these methods usually require training of a separate Gaussian process model and rely on the structural representation in latent space. There are other opportunities for improvement as well. Active learning (AL)^33,34^ attempts to expand the data set in areas where the ML model is most uncertain, which leads to more rapid model improvement. Another feature of AL is that it can employ physically meaningful dynamical trajectories for the sampling of configurations. In this Article we demonstrate how to keep these benefits of AL, while accelerating the rate of new data collection.

AL^33–35^ aims to iteratively collect diverse training data sets addressing any weaknesses identified in an ML model prediction. For this, it is necessary to estimate uncertainty for a model’s predictions^36–46^. A well-established practical strategy for AL with NN potentials is ‘query by committee’^47^ (QBC), where the estimate of uncertainty is the disagreement between a collection of models within an ensemble. Typically, there are five to ten NNs in an ensemble, and these share the same architecture and hyperparameters but, crucially, use a different initial randomization of the model parameters prior to training, as well as different splits of the training/validation data. It is empirically observed that the variance of the ensemble predictions correlates well with actual prediction error^37^, suggesting that the prediction task requires extrapolation beyond the range of the training data. In the QBC strategy, if this ensemble variance is observed to be large, then the training set will be augmented with new quantum simulation data.

AL estimates uncertainty in properties predicted for structures generated by an underlying sampler at each iteration. Molecular dynamics (MD) is the most popular method for sampling chemically meaningful potential energy surfaces. Without the uncertainty estimator, if we were simply running MD and picking random frames, even sampling at high temperature *T* would result in a lot of unnecessary DFT or other expensive ab initio data. The NN potential is not highly accurate at initial AL iterations. Thus, the resulting trajectory itself might be less physically relevant than the one obtained via DFT-based MD. However, as the NN potential improves during the AL procedure, the non-biased MD trajectory will be extremely similar to that obtained with DFT-MD. However, MD is susceptible to trapping in near-minimum conformations and only rarely enters chemically important regions such as transition states, which are key data for reactive simulations of chemical processes. In general, capturing thermodynamically rare events is a challenging task for any sampler. Metadynamics^48–51^ is an effective method of potential-energy-surface exploration, which operates on the concept of collective variables (CVs). However, CVs require manual selection, and their number is limited in practice. Choosing reasonable degrees of freedom to represent a reaction requires intuition and is a step where human error can produce errors. Furthermore, by choosing special degrees of freedom, the user has implicitly biased the kind of structures that will be sampled, potentially removing from the sampling space critical pathways. Therefore, this approach is not suitable for automatic sampling.

In this Article, following the idea of QBC and ensemble uncertainty, we propose an AL sampling algorithm biased towards regions of high uncertainty—uncertainty-driven dynamics (UDD). Due to model random initializations and the stochastic nature of training, regions of chemical space with low ensemble uncertainty will typically arise when similar regions are prevalent in the current training data set, such that every member of the ensemble is making an accurate inference. Thus, biasing MD in the direction of high ensemble uncertainty encourages the dynamics to visit new configurations, which are relevant for improving the diversity of the training set. Although the exact error may be under- or overpredicted by the ensemble uncertainty, the ensemble uncertainty will still identify relatively high-uncertainty structures to include in the AL data set, and UDD will still drive simulations towards these high-uncertainty structures. Biasing towards regions of large model uncertainty is also a strategy employed by Bayesian optimization^52,53^, which has been used successfully for the global optimization of atomic structures^54,55^. UDD-AL differs from Bayesian optimization in its intended purpose, which is to produce a diverse training data set of quantum simulation data; ML potentials trained to these data should generalize effectively to a wide range of chemical and configuration space.

One can regard the uncertainty-based bias potential as being similar to metadynamics in the sense that the sampling trajectories are pushed towards less-visited configurational regions. Here, however, CVs need not be defined. We show that within MD-based AL data acquisition, UDD helps substantially reduce the MD simulation time required to enter the high-uncertainty region. Most importantly, the proposed approach enables efficient conformational and configurational sampling at low-*T* conditions, making this approach essential for temperature-sensitive molecules. UDD assists in sampling the chemically relevant subspace of high-energy space, which contains important data such as transition states.

The value of the proposed approach is demonstrated here in two test cases. First, UDD-AL is used for conformational sampling of a glycine molecule. We find that the bias potential technique generates a diverse data set covering both low- and high-energy regions. As we show in the Results, this contrasts with high-*T* MD-AL, which tends to skip over low-energy regions. Second, in tests with acetylacetone at low-*T* conditions, the bias potential is observed to encourage the sampling of the phase space relevant to a proton transfer. Here we find that, in contrast with regular high-*T* MD, the bias potential technique encourages the reactive transition with very little distortion to the distribution of other degrees of freedom in the system.

## Results

### Uncertainty-driven dynamics for active learning (UDD-AL)

Before introducing UDD-AL, let us first set the context by reviewing the related method of metadynamics. Here, CVs *s*(*q*) are user-defined structural parameters being scanned by external Gaussian bias potentials. Usually, (3*N* − 6)-dimensional (where *N* is the number of atoms) atomic coordinates *q* of the simulated system are mapped to CVs, *s*(*q*). The corresponding energy function, ${E}_{{{{\mathrm{metadynamics}}}}}$, is defined as

1 $${E}_{{{{\mathrm{metadynamics}}}}}{(s,\,t)} = {\mathop {\sum}\limits_{{k\tau} < {t}} W (k\tau ){{{\mathrm{exp}}}}\left[ - \mathop {\sum }\limits_{i = 1}^{N_{{{{\mathrm{CV}}}}}} \frac{1}{{2b_i^2}}(s_i - s_i(q(k\tau )))^2\right]},$$

where *b*_*i*_ is the width of the Gaussian function for the *i*th collective variable, *W*(*kτ*) is the height of the Gaussian at the simulation time *t* = *kτ*, which is constant in the case of standard metadynamics, *τ* is the deposition rate of the Gaussian functions, *k* is the step number and *N*_CV_ is the number of CVs. During simulations, more Gaussians are added, thus discouraging the system to go back to its previous steps.

Like metadynamics, the UDD-AL method modifies the physical energy by adding a bias potential, *E*_bias_. Here, however, *E*_bias_ will be defined in terms of the model uncertainty rather than CVs. Such uncertainty estimates can be used to assist in the sampling of atomistic data^45^. In the QBC approach, an ensemble of NN potentials is trained, and the level of agreement between the NN predictions serves as the estimate of overall model uncertainty.

Here, as an argument of bias energy function, ${E}_{{{{\mathrm{bias}}}}}{\left( {\sigma _{\rm{E}}^2} \right)}$, we use the metric of ensemble disagreement, ${\sigma _{\rm{E}}^2}$, in the predicted energies, which is defined as

2 $${\sigma _{\rm{E}}^2} = {\frac{1}{2}\mathop {\sum }\limits_i^{N_{{{\mathrm{M}}}}} (\widehat{E_i} - {\hat{E}})^2},$$

where ${\widehat {E_i}}$ is the energy predicted by an ensemble member, ${{\hat{E}}}$ is its ensemble average, and *N*_M_ is the number of ensemble members (M), that is, NN potentials. Here, ensembles of ANI potentials are prepared using an eightfold cross-validation split of the data set, which yields *N*_M_ = 8 ensemble members (Methods). In previous applications of QBC-based AL, new data are collected when the uncertainty estimator *ρ* (standard deviation normalized by the square root of the number of atoms)

3 $${\rho} = {\sqrt {2/N_{\rm{M}}N_{\rm{A}}} \;\sigma _{\rm{E}}}$$

exceeds a threshold, where *N*_A_ is the number of atoms in a configuration. The justification of this metric is available in ref. ^37^.

Here we seek to construct a bias energy, ${E}_{{{{\mathrm{bias}}}}}{(\sigma _{\rm{E}}^2)}$, that favors configurations with larger uncertainties. ${\rho \propto \sigma _{\rm{E}}}$. Such configurations are expected to correlate with regions that are under-represented in the training data. A reasonable choice is the Gaussian function:

4 $${E}_{{{{\mathrm{bias}}}}}{\left( {\sigma _{\rm{E}}^2} \right)} = {A\left[ {{{{\mathrm{exp}}}}\left( { - \frac{{\sigma _{\rm{E}}^2}}{{N_{{{\mathrm{M}}}}N_{\rm{A}}B^2}}} \right) - 1} \right]}.$$

The magnitude *A* and width *B* of the biasing should be selected empirically. The bias potential goes to zero in the absence of uncertainty, *E*_bias_(0) = 0. Configurations with large uncertainty, ${\rho \gg B}$, are favored by a bias energy of magnitude *E*_bias_ ≈ −*A*. Forces derived from the bias potential are strongest when the uncertainty *ρ* is of the same order as the parameter *B*.

**Fig. 1:**
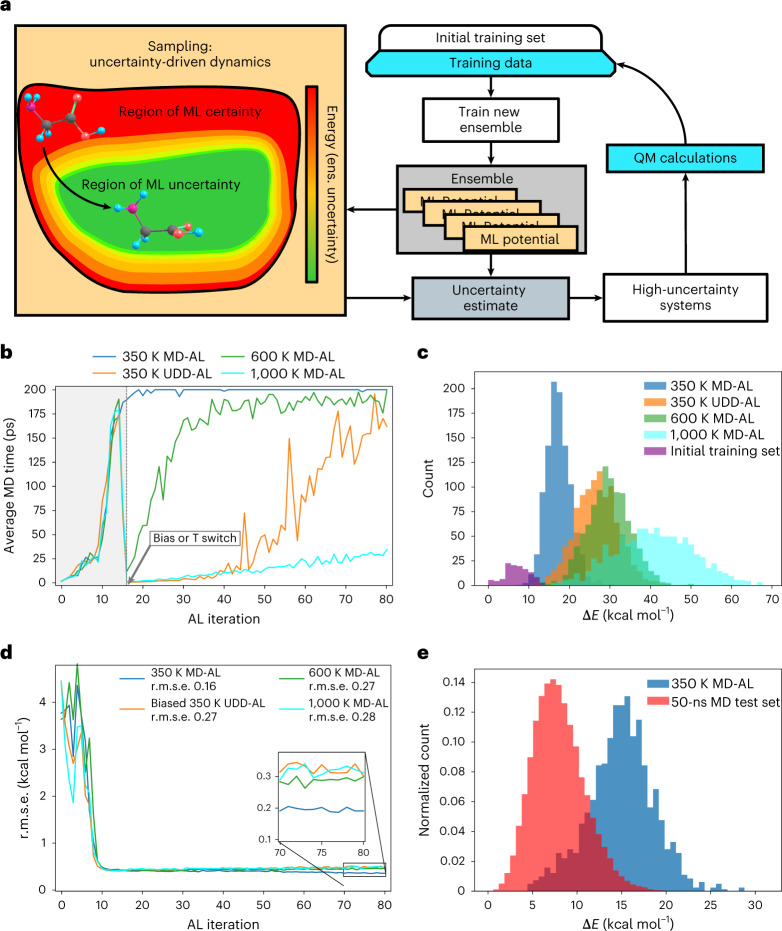
Comparison of UDD-AL and MD-AL approaches for a glycine test case. **a**, Schematic representation of the UDD-AL workflow. ens., ensemble. **b**, Average MD time required to meet the uncertainty criterion versus AL iteration for four different MD simulation types: 350 K MD-AL, blue; 600 K MD-AL, green; 1,000 K MD-AL, cyan; 350 K UDD-AL, orange. **c**, Energy distribution histograms of four data sets sampled by the 350 K MD-AL (blue), 600 K AL MD-AL (green), 1,000 K MD-AL (cyan) and 350 K UDD-AL (orange). Data from iterations 0–14 are omitted because the bias energy term is turned off, or the temperature is not increased at this stage. **d**, Comparison of potential energy r.m.s.e. obtained on the 50-ns test set versus AL iteration (that is, training set size, 16 new glycine conformations per iteration). The legend shows the r.m.s.e. for models trained on data from the entire AL procedure. **e**, Normalized energy distribution histograms of the 50-ns test set (red) and training set sampled by 350 K MD-AL (blue). Lines in **b** and **d** are averaged over three ensembles, each trained on data from an independent AL procedure. Source data

The combined potential ${{\hat{E}} + E_{{{{\mathrm{bias}}}}}}$ is used to define a UDD. In applications to AL, the overall strategy will be denoted UDD-AL. The schematic workflow of UDD-AL is depicted in Fig. 1a. It should be compared to the usual MD-based approach (MD-AL), which does not incorporate the *E*_bias_ term.

Determining reasonable values for *A* and *B* is an important preliminary step for the UDD approach. Although the method is fairly insensitive to *A* and *B*, extremely poor choices of *A* and *B* can render the approach less efficient. The optimal parameters *A* and *B* will be context-dependent. For example, our applications to glycine and acetylacetone tests, discussed below, suggest that the bias magnitude *A* should be at least of the order of the energy barriers of interest. The bias width *B* can be selected by considering the magnitude of the bias force contribution. When *B* is too low, the bias forces are very large and disrupt the dynamics completely. When *B* is too large, the bias potential is very smooth and does not play a large role in the dynamics. We selected *B* empirically and found that the ratio *r* (equation (5)) of bias force magnitude to true interatomic force magnitude on the selected samples during the initial iterations (1st to 14th in the case of glycine) of MD-AL is ~0.36:

5 $${r} = {\frac{1}{{N_{\rm{S}}N_{\rm{A}}}}\mathop {\sum }\limits_I^{\rm{Structures}} \mathop {\sum }\limits_J^{\rm{Atoms}} \frac{{||F_{IJ}^{\rm{bias}}||}}{{||F_{IJ}^{\rm{true}}||}}}.$$

Here, *N*_S_ is the number of structures collected during the initial iterations, *N*_A_ is the number of atoms, ${F}_{IJ}^{\,\rm{bias}}$ is the bias force acting on atom *J* in structure *I*, and ${F}_{IJ}^{\,\rm{true}}$ is the interatomic force acting on atom *J* in structure *I*. We thus suggest that *B* should be selected to approximately replicate this ratio on data selected by an initial phase of MD-AL.

The effective bias forces on an atom at position *r* can be calculated using the chain rule

6 $${- \frac{\partial }{{\partial r}}E_{{{{\mathrm{bias}}}}}(\sigma _{\rm{E}}^2)} = {- E_{{{{\mathrm{bias}}}}}(\sigma _{\rm{E}}^2)^\prime \frac{\partial }{{\partial r}}\sigma _{\rm{E}}^2}$$

and

7 $${- \frac{\partial }{{\partial r}}\sigma _{\rm{E}}^2} = {- \mathop {\sum }\limits_i^M ({\widehat{E_i}} - {\hat{E}})\frac{\partial }{{\partial {r}}}({\widehat{E_i}} - {\hat{E}})} = {\mathop {\sum }\limits_i^M ({\widehat{E_i}} - {\hat{E}})(\,{\widehat{f_i}} - {\hat{f}}\,)},$$

where ${\widehat {f_i}}$ denotes the force vector predicted by an ensemble member, and ${{\hat{f}}}$ is the ensemble-averaged prediction.

### Glycine conformational space sampling

The glycine molecule is shown in Fig. 1a. We are interested in sampling the conformational space without bond-breaking events, using various AL protocols. As shown in ref. ^56^, this smallest amino acid is a challenging data acquisition task because of the various conformational minima on its potential energy surface. Dihedral rotations of –NH_2_ and –COOH groups correspond to barriers of 2.5–3.5 kcal mol^−1^, depending on the initial conformation. Our numerical tests have shown that a bias magnitude *A* that is approximately five times higher than the average barrier of interest provides the best results for the glycine test. It helps keep the bias potential effectively high at higher uncertainty values, since it decays exponentially with the uncertainty increase. It also helps overcome possible barrier bottlenecks caused by overestimation of the barrier heights by the ML model. The geometries near the global energy minimum (GM) of glycine are already present in the initial training set, and each sampling MD simulation starts with this kind of structure. Depending on the AL iteration, near-GM structures have a value of *ρ* of $0.024 \pm 0.005 \, {{{{{\mathrm{kcal}}}} \times {{{\mathrm{mol}}}}^{ - 1} \times { {N_{\rm{A}}} } ^{ - 1/2}}}$ (*σ*_E_ = 0.15 ± 0.03 kcal mol^−1^). We thus selected *A* equal to 15.4 kcal mol^−1^, which corresponds to a bias potential of ~15.0 kcal mol^−1^ at near-GM values of *ρ* (with *B* = 0.12 kcal mol^−1^). We further increased *A* by 15% at the 140-ps time step if the uncertainty criterion was not met at this simulation stage. Our tests also show that the best results are achieved for *B* close to the near-GM uncertainty, *σ*_E_. Here we use *B* = 0.12 kcal mol^−1^ for glycine.

**Fig. 2:**
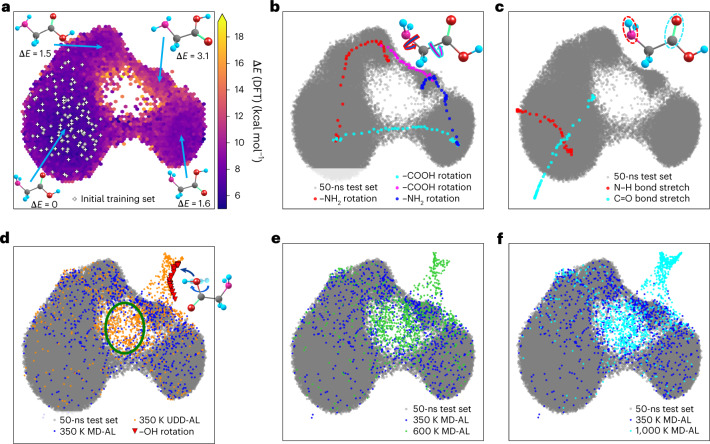
Two-dimensional representation of the glycine conformational space processed by the UMAP dimensionality-reduction technique. **a**, The 50-ns test set. The heat map represents the relative DFT energy. Glycine insets denote the corresponding conformational region. Atom colors: H, blue; C, gray; N, pink; O, red. **b**–**d**, Data sets and scans are placed over the 50-ns test set (gray). **b**, Conformational paths through –COOH (cyan and purple) and –NH_2_ (red and dark blue) rotations. **c**, N–H (red) and C=O (cyan) bond length scans. **d**, Comparison of training sets sampled by 350 K MD-AL (blue) and 350 K UDD-AL (orange). The green oval denotes the inner high-energy region. Red triangles denote the scan of the –OH rotation around the C–O bond. **e**, Comparison of training sets sampled by 350 K MD-AL (blue) and 600 K MD-AL (green). **f**, Comparison of training sets sampled by 350 K MD-AL (blue) and 1,000 K MD-AL (cyan). Source data

We next compare the two AL approaches—UDD-AL and MD-AL—for the task of collecting a data set of the glycine conformational space. Each AL iteration performs 16 MD simulations with a 200-ps time limit and 1-fs steps (MD simulations section). An ensemble of NN potentials for the first AL iteration is trained on the initial data set of 125 conformers, spanning the near-equilibrium structures of the glycine GM. At each subsequent AL iteration, the MD simulation employs an ensemble of ANI-type NN potentials (Active learning section and ref. ^57^), trained on the initial data and data accumulated on all previous AL iterations. The starting geometries for the MD simulations and the initial training set contain only near-equilibrium geometries of a glycine GM (Fig. 2a). Stated differently, NNs have no initial information about higher-energy conformers, and the MD simulations have to reach them from the bottom of the potential energy surface. Each MD simulation is terminated when the system meets the uncertainty selection criterion *ρ* of $0.35 \, {{{{{\mathrm{kcal}}}} \times {{{\mathrm{mol}}}}^{ - 1} \times {{N_{\rm{A}}} } ^{ - 1/2}}}$ (Active learning section). If the MD simulation reaches the time limit, then the structure from the trajectory with the highest uncertainty is selected. DFT reference data (MD simulations section) are then computed for the final conformations and added to the training set for the next iteration of the AL process.

Figure 1b shows the average MD simulation time required to meet the uncertainty criterion in different AL approaches with respect to AL iteration. The MD-AL at low-*T* conditions (350 K) reaches the MD time limit at approximately the 20th iteration, and is continued until the final AL iteration (Fig. 1b, dark blue line). This means that the specified uncertainty criterion is almost never met, and the sampler returns the geometry of the maximum available uncertainty from the MD trajectory. The uncertainty bias potential is introduced in low-*T* (350 K) MD simulations (orange line). We do not activate the bias potential at earlier AL iterations for two reasons. First, the low-*T* MD-AL (Fig. 1b, dark blue line) does not reach the MD simulation time limit up to the 15th to 20th iterations. Thus, at this stage, the regular MD-AL manages to acquire new data satisfying the uncertainty criterion. Second, the NN potential might be unstable or not smooth at earlier iterations due to the lack of data. Therefore, the bias is activated at the 15th iteration to avoid moving systems towards unphysical configurations. When the uncertainty bias potential is on (the UDD-AL regime), it reduces the number of MD steps needed to meet the desired uncertainty. Moreover, the MD time limit plateau is still not reached until the final iteration.

Perhaps the most common way to accelerate sampling of high-energy states is to run high-*T* MD. Thus, to illustrate the difference between the bias potential and a simple temperature increase, we also compared the low-*T* 350 K UDD-AL with the high-*T* MD-ALs at 600 K and 1,000 K simulation conditions (Fig. 1b). As in the case of UDD-AL sampling, the temperature is increased at the 15th iteration. The 600 K MD-AL approaches the MD time limit at approximately the 40th iteration, whereas UDD-AL reaches the time limit plateau by the end of the AL procedure. Thus, 600 K MD-AL does not perform as well as UDD-AL in terms of simulation time. On the other hand, the 1,000 K MD-AL (cyan line) exhibits faster sampling, and the average MD time does not exceed 35 ps, even at the last AL iteration.

The energy ranges sampled by each AL approach are shown in Fig. 1c. As expected, the high-*T* MD-AL data (green and cyan histograms) span wider energy ranges than the low-*T* 350 K MD-AL (blue histogram). What is interesting is that the energy distribution of data from the 350 K UDD-AL (orange histogram) is very similar to the shape of the 600 K MD-AL data. An advantage of UDD-AL is that many fewer MD steps are required to collect these samples.

After completing the entire AL procedure, the final models are trained on 1,280 glycine conformers collected during the procedure (+125 conformers in the initial training set). To access the accuracy of the four models, we use a test set of 50,000 glycine structures from a 50-ns MD simulation via an ANI-1ccx potential run at 400 K with a 0.5-fs time step^19^. The relatively low *T* of 400 K for this test set was chosen to effectively sample the low-energy conformational space of glycine. Here, we aim to test whether low-temperature 350 K UDD-AL can not only sample the same low-energy space, but also sample higher-energy conformers in the same temperature regime. The difference between 350 K and 400 K temperatures arises from the fact that we want to speed up the generation of the 50-ns test set without a substantial change in the population of conformers. As depicted in Fig. 1d, all models perform reasonably well, with root-mean-square error (r.m.s.e.) of less than 0.3 kcal mol^−1^. However, the r.m.s.e. of the model trained on low-*T* 350 K MD-AL data is slightly, yet systematically, lower than the r.m.s.e. for the other models, with a difference of ~0.11 kcal mol^−1^. This is probably because MD simulations tend to oscillate in near-equilibrium positions, which is why this test set is dominated by low-energy geometries. The low-*T* 350 K MD-AL, in turn, densely spans a narrow low-energy range, which might explain the slightly better performance of this model on a test set derived from a 50-ns MD. Indeed, the normalized histograms in Fig. 1e show that this seemingly large 50-ns test set spans the energy region closest (but even lower) to the one covered by the low-*T* 350 K MD-AL data.

**Extended Data Fig. 1:**
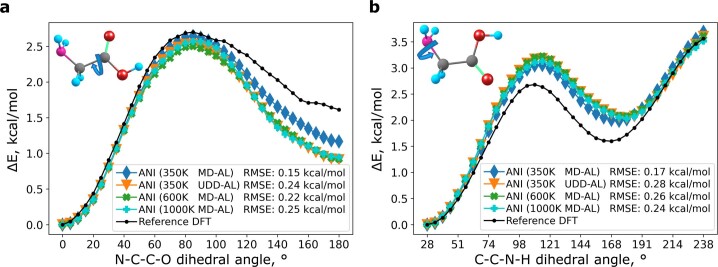
Models’ performance on low-energy dihedral rotation profiles. **a** A relaxed potential energy scan of dihedral -COOH rotation in glycine generated from reference DFT (black) and models trained on 350 K MD-AL (blue), 600 K MD-AL (green), 1000 K MD-AL (cyan), and 350 K UDD-AL (orange) data. Each figure also lists the RMSE, for each model compared to the reference DFT method. **b** Same as **a** but potential energy scan of dihedral -NH_2_ rotation. Atom colors: H – blue, C – gray, N – pink, O – red. Source data

Note that the 50-ns test set is a MD trajectory with no AL involved, whereas the MD-AL data comprise structures with high uncertainties. High uncertainty usually corresponds to a higher energy due to poor sampling in normal low-*T* MD. In other words, the 50-ns MD test set is biased towards near-equilibrium oscillations, but the MD-AL data are selectively augmented with higher-energy isomers. A similar r.m.s.e. shift is observed for low-energy rotations of the –COOH and –NH_2_ functional groups (Extended Data Fig. 1). Accordingly, the various models should also be tested on higher-energy pathways.

To illustrate what types of chemical process appear in the system, and how each sampling method covers them, we next visualize the glycine conformational space using dimensionality reduction. We project samples to a two-dimensional (2D) plane using the Uniform Manifold Approximation and Projection (UMAP)^58^ technique, where each conformation is characterized by the 672-dimensional vector of activations (concatenated atomic environment vectors) in the first layer of an independent pre-trained ANI-1x model^19^. Figure 2a shows the 50-ns MD data set spanning four low-lying glycine conformers represented by four regions in 2D space. Torsional conformations, a N–H bond scan and a C=O bond scan are depicted in Fig. 2b,c to illustrate the structural profiles in 2D space.

Figure 2d–f depicts the four data sets visualized over the 50-ns test set. Figure 2d provides a visual comparison of data sampled by MD-AL and UDD-AL at 350 K. Both data sets cover the 50-ns MD data reasonably well. However, there are two key differences. First, a high-energy configurational space (points inside the green oval in Fig. 2d) is more densely sampled in the UDD-AL data set. There are 289 points in this non-equilibrium region in the UDD-AL sampling, compared to 105 points in the 350 K MD-AL. Second, UDD-AL encountered a new conformational path in the top right corner of Fig. 2d that was not accessed by 350 K MD-AL. This region corresponds to rotation of the –OH group around the C–O bond, which is a distinct conformational transition, and a high-energy profile with a barrier of 15 kcal mol^−1^.

Figure 2e presents a visual comparison of the sampling performance of MD-ALs at 350 K and 600 K. The 2D representation of 600 K data is quite similar to the one for the 350 K UDD-AL data: there are 290 data points inside the inner circled region and a good coverage of the –OH rotation region. As expected, MD-AL at the extreme temperature of 1,000 K (Fig. 2f) samples the inner high-energy region even more densely (394 data points), as well as the –OH rotation region. This, however, comes at a cost. The low-energy region in the lower left of Fig. 2f clearly demonstrates a lack of sampling. This is the primary deficiency of using high-*T* MD: as the temperature increases, the system spends less time near low-energy regions, because in these regions the kinetic energy is typically the greatest. It will therefore be possible to ‘skip over’ regions of high stability, thus resulting in a poor data coverage of the near-equilibrium region. On the other hand, UDD-AL sampling does not run this risk, by sufficiently sampling any relevant region.

Accordingly, Fig. 2 shows that the UDD-AL is an efficient, balanced way of sampling the chemical space, reaching most of the high-energy points achieved with 1,000 K MD sampling, but without losing data density in low-energy regions. However, alone, it is not clear that biased sampling presents advantages over unbiased high-*T* sampling; UDD-AL appears to sample similar configurations to the 600 K MD-AL. Therefore, we performed additional tests of high-energy pathways that illuminate the differences between the 600 K MD-AL and 350 K UDD-AL. A discussion of the high-energy profiles—angle and bond scans—is provided in Supplementary Section 2. The overall trend is that the 350 K UDD-AL model exhibits much better accuracy than the model trained on low-*T* 350 K MD-AL data. When comparing the UDD-AL with 600 K and 1,000 K MD-AL models, the former results in better, or at least comparable, accuracy.

In Supplementary Section 3 we also provide a detailed overall assessment of the performance of the sampling strategy by cross-testing the associated models on the data from all sampling strategies. Supplementary Table 2 summarizes the r.m.s.e. values of the four models on the test sets accumulated by each AL sampler: 350 K MD-AL, 350 K UDD-AL, 600 K MD-AL and 1,000 K MD-AL. When testing models on data sets that are not generated by the same corresponding sampler, the UDD-AL model outperforms all other models.

**Fig. 3:**
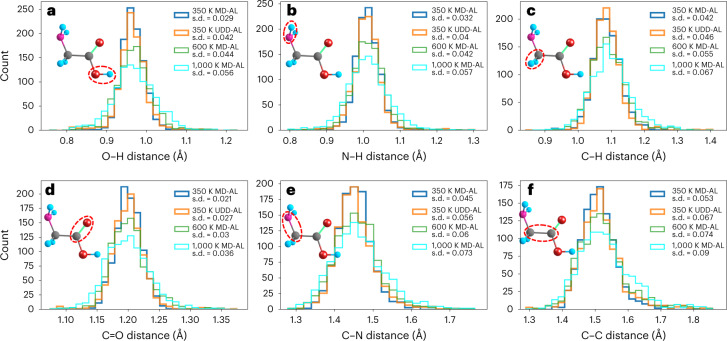
Glycine interatomic distance distributions in the MD-AL and UDD-AL data sets. **a**–**f**, Comparisons of bond length distributions in the 350 K MD-AL (blue), 350 K UDD-AL (orange), 600 K MD-AL (green) and 1,000 K MD-AL (cyan) data sets for O–H (**a**), N–H (**b**), C–H (**c**), C=O (**d**), C–N (**e**) and C–C (**f**). Bond length standard deviations (s.d.) are indicated in the legend. The bond of interest is highlighted with a red dashed oval. Source data

Ultimately, when looking at a variety of bond rotations and stretches, the most accurate energy profile changes depending on the energy range of the specific scan. Low-energy profiles tend to be modeled better by the low-*T* data set, whereas higher-energy scans are accessed better by the higher-*T* data set. However, the UDD-AL sampling method yields a model that performs well on a wide range of energy profiles, while also maintaining a low error on the held-out test set for each sampling method (Supplementary Table 2). This difference suggests that UDD-AL is able to avoid the higher-energy and less chemically relevant structural distortions, which are typical at very high temperatures. Meanwhile, chemically relevant structures present in the UDD-AL data set enable efficient extrapolation to the higher-energy structures present in the 1,000 K MD-AL data. As can be seen in Fig. 3, the shapes of the interatomic distance distributions in UDD-AL closely mimic the sharp distributions in low-*T* 350 K MD-AL, although with a larger standard deviation. This deviation, however, is lower than in the 600 K and 1,000 K MD-AL data sets, which span a wider distance range. This, in turn, further suggests that the UDD sampler tends to avoid random distortions found in high-*T* regimes.

### Proton transfer in acetylacetone

**Fig. 4:**
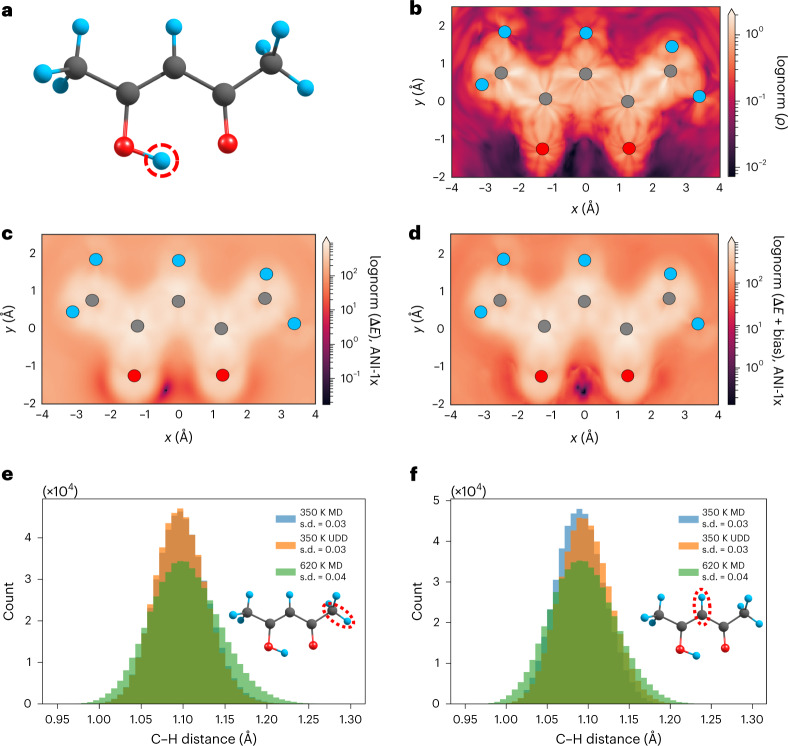
Ensemble uncertainty and UDD in acetylacetone. **a**, Acetylacetone molecule. Atom colors: H, blue; C, gray; N, pink; O, red. The same key applies to the molecules in **b**–**f**. The red dashed circle denotes the hydrogen atom involved in a proton transfer. **b**, Log-normalized map of uncertainty *ρ* of the ANI-1x model ensemble with respect to the position of a circled hydrogen. **c**, Log-normalized map of physical energy. **d**, Log-normalized map of summed physical and bias energy. **e**,**f**, Comparisons of C–H bond length distributions (corresponding bonds are indicated by a red dashed ovals) in methyl (**e**) and central (**f**) groups, for 350 K MD (blue), 350 K UDD (orange) and 620 K MD (green) simulations. Red ellipses denote the bond under consideration. Each subplot also lists the bond length s.d. from the equilibrium distance per legend. Source data

We further examine the performance and transferability of UDD for sampling of a reactive pathway in a larger molecule, an acetylacetone enol tautomer^59^, as depicted in Fig. 4a. We are interested, in particular, in the proton transfer between the two oxygen atoms, considering the proton position as a free variable. Instead of using AL techniques, here we use an ensemble of pre-trained ANI-1x interatomic potentials^19^, which were not trained on bond-breaking reactions, and analyze trajectories from UDD and MD simulations. ANI-1x was trained at the wB97x DFT level of theory, yielding a barrier of 4.7 kcal mol^−1^. However, ANI-1x overestimates the barrier, giving a value of 6.3 kcal mol^−1^. This value is selected as bias magnitude *A*. Uncertainty values, *ρ*, of the near-equilibrium acetylacetone structures within the ANI-1x model are higher by an order of magnitude than those produced by the newly trained model for glycine. Accordingly, we set a higher value of bias width *B* = 0.45 kcal mol^−1^, this being an empirically adjusted parameter.

Figure 4b presents the log-normalized uncertainty *ρ* of the acetylacetone system with respect to the position of the proton. The dark region (near *x* = 0 Å and *y* = −1.5 Å) demonstrates that there is a high-uncertainty region between oxygen atoms that corresponds to a proton transfer transition state. Figure 4c depicts the log-normalized relative potential energy (ANI-1x) of the system with respect to the position of the free proton. The dark region (near *x* = −0.5 Å and *y* = −1.5 Å) indicates that the lowest energy corresponds to a proton position near the oxygen atom. This is an expected result, because the hydrogen bound to the oxygen atom is the most stable geometry. However, as shown in Fig. 4d, the energy minimum can be shifted to the central position between oxygen atoms (dark region near *x* = 0 Å) when a bias potential is applied. For illustrative purposes, here we use a high value of bias magnitude *A* = 56.0 kcal mol^−1^. In practice, we use *A* = 6.3 kcal mol^−1^ for the MD simulation discussed below.

**Extended Data Fig. 2:**
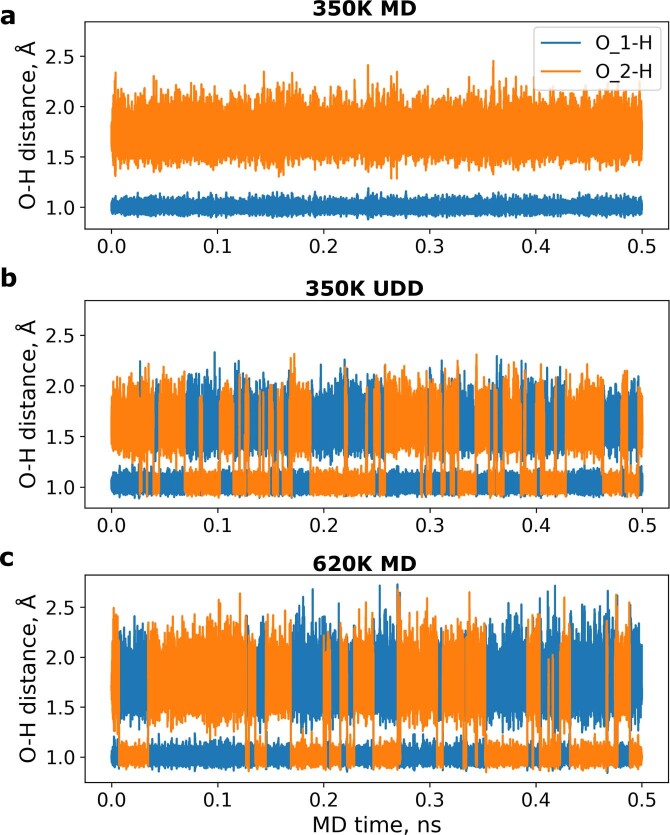
Comparison of proton transfer rates in regular MD and UDD simulations. Each subplot shows two O-H distances (blue and orange) in the acetylacetone molecule throughout the 0.5 ns MD simulation. **a** Regular 350 K MD. The legend corresponds to all three panels. **b** 350 K UDD (uncertainty-based bias potential applied). **c** Regular 620 K MD. Source data

We also analyzed the results from 0.5-ns trajectories obtained using UDD and regular MD simulation techniques. No proton transfer occurs during the regular 350 K MD simulation. Meanwhile, the uncertainty bias can direct the proton towards a high-uncertainty region between two oxygen atoms (90 proton transitions are observed in the 350 K UDD simulation). Finally, the unbiased high-*T* 620 K MD results in 48 proton transitions. Although at a lower rate compared to UDD, the increased temperature also facilitates proton transfer. Time traces of the two O–H distances are provided in Extended Data Fig. 2.

A key difference between low-*T* UDD and regular high-*T* MDs can be observed when analyzing the oscillations of interatomic distances. High-*T* conditions will affect the entire molecule, causing larger distance deviations compared to low-*T* conditions. Indeed, the overall spread of O–H distances is comparable when using 350 K MD (Extended Data Fig. 2), but far wider with 620 K MD, even in segments of the trajectory without proton transfer. Further analysis of C–H distances in the molecule, shown in Fig. 4e,f, confirms this phenomenon. Figure 4e shows C–H distance distributions in the methyl group in 350 K MD, 350 K UDD and 620 K MD simulations. The 620 K MD exhibits higher deviations from the equilibrium C–H bond length compared to low-*T* UDD. The standard deviation of the methyl C–H distance in 620 K MD is 0.04 Å, versus 0.03 Å with 350 K UDD. Notably, the low-*T* 350 K MD trajectory has a standard deviation of 0.03 Å, the same as for low-*T* UDD. The same picture holds for the central C–H bond described in Fig. 4f.

These observations confirm that, although high-*T* sampling promotes the activation of reactive pathways, it has a global effect on all degrees of freedom in the system, whereas the UDD technique allows us to sample the reactive pathway without substantial changes to the equilibrium distributions of other degrees of freedom. This is probably due to the composition of the ANI-1x training data, which has information on non-equilibrium extended bonds. However, a hydrogen that is equidistant between two oxygen atoms is not commonly encountered in configurational data. Thus, the UDD potential promotes sampling of this specific region.

## Discussion

The key advantage of UDD-AL over regular high-*T* sampling is that UDD-AL facilitates the sampling of important under-represented chemical data, without the random structural distortions caused by high-*T* conditions. This feature can be used for efficient sampling of the conformational and/or configurational space of temperature-sensitive or metastable systems. Our tests also indicate that the bias potential can facilitate sampling of high-energy chemical space, without sacrificing the sampling of low-energy configurations. This means that UDD will produce robust data sets that are applicable to both lower-energy, near-GM data and high-energy chemical space, which usually corresponds to important reactive structural data such as transition states and intermediates. One topic of future research could be the interface of the ML potential trained on UDD-AL data with weighted ensemble methods for obtaining the pathways and rates of chemical reactions.

According to equations (4) and (7), including the bias potential has a negligible effect on the computational time, because ${\sigma _{\rm{E}}^2}$, ${\widehat {E_i}}$ and ${\widehat {f_i}}$ are calculated at each MD step in the ensemble-based AL. This makes the proposed approach applicable to a wide range of chemical systems, for example, for modeling larger molecules and bulk systems. It is possible that larger systems would also benefit from the application of uncertainty bias derived from force predictions.

In the glycine test case, because all the models used the same hyperparameters, it is possible that each model could perform better if individual hyperparameter searches were carried out. Data sets that cover a broader chemical space may need more learnable parameters to be flexible enough to fit the effectively larger degrees of freedom to which they are being trained. This will be a subject of future studies.

As the results show, the uncertainty-based bias potential is a promising technique for sampling rare events while being relatively faithful to the physical equilibrium distribution. The UDD is similar to metadynamics^48–51^ in its use of a bias potential. However, one remarkable advantage of UDD compared to metadynamics is that UDD avoids the need to manually select CVs or to identify basins of attraction, which require a great deal of domain expertise and trial and error. In a way, it defines the best CV for the purpose of AL: training a more general and robust ML potential. The major limitation of UDD-AL is that the approach requires selection of two parameters: the bias magnitude and width. In this Article, these parameters are context-dependent and selected based on the height of barriers of interest and the ratio of bias/true interatomic forces. However, developing a method that can tune these algorithmically would be a productive future activity. Perhaps this problem could be reduced to the selection of just one parameter—bias magnitude—when using a linear bias function rather than exponential. Additionally, an algorithm for automatic selection of uncertainty criteria could improve the sampling efficiency.

After the completion of this study, we became aware of a closely related work^60^. In that study, the UDD is interfaced with the Atomic Cluster Expansion model. The authors achieved a good sampling of AlSi_10_ and polyethylene glycol, which highlights the versatility of the UDD-AL approach across ML methods and chemical compositions.

## Methods

### Active learning

For the glycine simulations, we used the ANI deep learning model^57^ to generate ensembles of NN potentials prepared using an eightfold cross-validation split of the data set. The empirical value of $0.23 \, {{{{{\mathrm{kcal}}}} \times {{{\mathrm{mol}}}}^{ - 1} \times {{N_{\rm{A}}} } ^{ - 1/2}}}$ for the uncertainty selection criterion *ρ*, equation (3), provided in the original work on AL for organic molecules^37^, turned out to be too low for the purposes of training on one chemical system. It causes unnecessarily dense sampling of the glycine conformational space, which, in turn, hinders the MD simulation in reaching higher-energy regions. We thus used a higher value of $0.35 \, {{{{{\mathrm{kcal}}}} \times {{{\mathrm{mol}}}}^{ - 1} \times { {N_{\rm{A}}} }^{ - 1/2}}}$ for this test case. Overall, automatic selection of uncertainty criteria is a non-trivial question that deserves a separate discussion and goes beyond the scope of this work. Each MD simulation was terminated when the system met the uncertainty selection criterion *ρ*.

The initial training set consisted of 125 glycine geometries that span the near-equilibrium structures of the glycine GM. These data were acquired from a separate 5-ps MD trajectory at 350 K with a 0.5-fs time step, initialized from the glycine GM. Every 80th MD step was included in the initial data training set. The MD simulation for the initial training set was carried out using the pre-trained ANI-1x potential^57^.

### MD simulations

In all discussed cases, the Langevin thermostat was used to maintain temperature, with a friction coefficient of 0.01 a.u. Each AL iteration performed 16 MD simulations with a 1-fs time step and a limit of 200,000 steps (200 ps). At each AL iteration, the MD was driven by an ensemble of ANI-type ML potentials, trained on initial data and data accumulated on previous AL iterations. The NN-based MD was interfaced with Atomic Simulation Environment code^61^. The final data set had 1,280 data points sampled in the AL procedure and 125 data points from the initial data set.

The set of seed geometries for MD simulations comprised 25 structures that corresponded to near-equilibrium geometries of a glycine GM. These were selected as the first 25 structures from the initial training set. The AL sampler randomly selected one of them for each MD initialization. The energies and forces of the new conformers were calculated using the ωB97X-D/cc-pVTZ^62,63^ level of theory, as implemented in PSI4 code^64^.

### NN architecture

The parameters for the atomic environment vector^57^ (a numerical vector used to encode the atomic local environment in ANI) used during the AL process were constant. Thirty-two evenly spaced shifting parameters were used for the radial part of the vector, with a 4.6-Å cutoff radius, and a total of eight radial and eight angular shifting parameters were used for the angular part, with a 3.5-Å cutoff radius. With four atom types, this gave 768 elements in the descriptor. The first atom-centered function was shifted to 0.8 Å from the atomic center. The ANI potential used in this work contained three hidden layers and had the architecture 768:32:16:8:1, where each number describes the number of neurons at each subsequent layer in the network.

The ANI potential used in this work contained three hidden layers and had the following architecture: 768:32:16:8:1. Gaussian activation functions were used in the hidden layers and linear activation in the final layer.

The data set was split in the ratio 14:1:1 for training, validation and testing. An initial learning rate of 0.001 was used with a batch size of 32. Early stopping was utilized in the training of each network, such that if a model failed to improve its validation set predictions within 50 epochs, then training was stopped. Learning rate annealing was utilized, so that if a model stopped early, training was restarted with a learning rate 0.5 times that of the previous learning rate. Termination of training was achieved when the learning rate was less than 10^−5^. The Adam^65^ optimizer was used to update the weights during training. More details on ANI-type potentials and the AL technique are provided in refs. ^19,37,54^.

For the final models, the average number of training epochs was 695 on NVIDIA TITAN V GPUs with an average of 0.098 s per epoch. For acetylacetone MD simulations, a pre-trained ANI-1x model was used^19^.

A summary of training and prediction timings is provided in Supplementary Section 1.

### Supplementary information

Supplementary Information Supplementary Sections 1–3, Figs. 1–4, Tables 1 and 2 and discussion.

### Source data

Source Data Fig. 1 Statistical source data for Fig. 1.

Source Data Fig. 2 Statistical source data for Fig. 2.

Source Data Fig. 3 Statistical source data for Fig. 3.

Source Data Fig. 4 Statistical source data for Fig. 4.

Source Data Extended Data Fig. 1 Statistical source data for Extended Data Fig. 1.

Source Data Extended Data Fig. 2 Statistical source data for Extended Data Fig. 2.

## Data Availability

All data generated or analyzed during this study are available at 10.5281/zenodo.7526389 (ref. ^66^). Source data are provided with this paper.

## Code availability

Compiled standalone software and source code for reproducing the analyses and figures in this paper, as well as for applying the methodology we have developed here, are freely available at https://github.com/lanl/Active-Learning-Framework/tree/udd-al and 10.5281/zenodo.7522399 (ref. ^67^).
