# Supplementary material for: Uncertainty-driven dynamics for active learning of interatomic potentials
Source: Nat Comput Sci. 2023 Mar 6;3(3):230–9. doi: 10.1038/s43588-023-00406-5 (PMC10766548; doi:10.1038/s43588-023-00406-5)
Supplement: Supplementary file 1 — Supplementary Sections 1–3, Figs. 1–4, Tables 1 and 2 and discussion. [file 43588_2023_406_MOESM1_ESM.pdf]

nature computational science

Article https://doi.org/10.1038/s43588-023-00406-5

Uncertainty-driven dynamics for active learning of interatomic potentials

In the format provided by the authors and unedited

Supplementary Information

Supplementary Section 1: NN timing

Supplementary Table 1: Energy and force prediction timing (on Intel Xeon Gold 6138 CPU)

System	# of atoms	Time (ms) per configuration
Glycine	10	2.5 (Newly trained ANI)
Acetylacetone	15	2.7 (ANI-1x)

Supplementary Section 2: Analysis of higher energy profiles

The first choice of high-energy test case is the –OH rotation around the C-O bond with a 15 kcal/mol barrier, which was evidently sampled better by high-T MD-AL and low-T UDD-AL according to Fig.2 (in main text). Indeed, as we can see in Supplementary Figure 1a (top), the model trained on low-T MD-AL data (blue) significantly deviates from the reference DFT data (black) with the total RMSE of 13.8 kcal/mol. The model trained on low-T UDD-AL data (orange), in turn, exhibits excellent quantitative agreement with the DFT profile having the RMSE of 0.8 kcal/mol. This is also reflected by the disagreement plot in Supplementary Figure 1a (bottom) where the model trained on low-T MD-AL data has large ρ values at the angle > 80° while the model trained on UDD-AL data is certain over the entire transition path. Models trained on high-T (600K and 1000K) MD-AL data sets (green and cyan) also agree well with the DFT data.

In Supplementary Figure 1b, we examine the models' accuracy on an even higher energy profile, C-C-N angle bend, which has 80 kcal/mol relative energy at the 180° bend coordinate. Notably, here the 600K MD-AL model performs substantially worse at the highest angles compared to the UDD-AL model having RMSE of 4.5 *vs.* 1.5 kcal/mol. This observation becomes even more interesting when bearing in mind the fact that 350K UDD-Al and regular 600K MD-AL training sets result in nearly the same UMAP plots (Figures 2d and 2e in main text). Moreover, according to the histogram plot in Fig.1b (main text), the 600K MD-AL training set has a higher mean energy of 22.0 kcal/mol *vs.* 19.1 kcal/mol in 350K UDD-Al training data. Meanwhile, the model trained on 1000K MD-AL data outperforms the UDD-AL model on a C-C-N bend profile. Therefore, the minimum sampling temperature required for accurate prediction of C-C-N bend energies lies somewhere in the range 600-1000K for the regular MD-AL approach. At the same time, biased UDD-AL handles this task at a relatively low 350K.

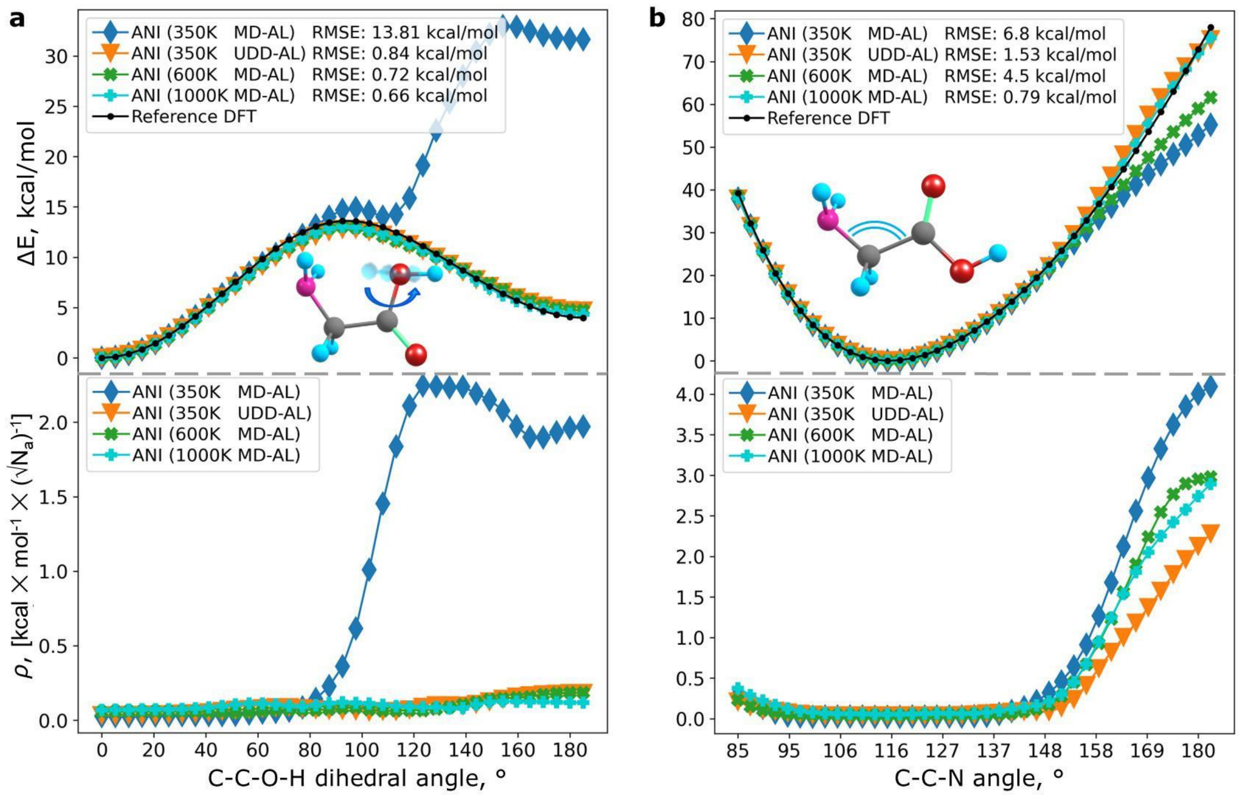

Supplementary Figure 1 | Models' performance on angle scans. Each subplot shows a relaxed potential surface scan generated from reference DFT and models trained on 350K MD-AL (blue), 600K MD-AL (green), 1000K (cyan) MD-AL, and 350K UDD-AL (orange) data. Each line is averaged over three ensembles, each trained on data from an independent AL procedure. **a** Potential energy scan of -OH rotation around the C-O bond (top) and the corresponding ensemble uncertainty (bottom). Here, the O-C-C-N dihedral angle is fixed. **b** Potential energy scan of C-C-N angle bend (top) and the corresponding ensemble uncertainty (bottom). Atom colors: H – blue, C – grey, N – pink, O – red.

In Supplementary Figure 2, we examine the models' performances on bond length scans. For the N-H length in Supplementary Figure 2a, the model trained on low-T MD-AL data fails to extrapolate to conformations with extreme stretch coordinates. At the same time, both models trained on 350K UDD-AL and 600K/1000K MD-AL data successfully reproduce DFT energies at boundary bond length regions, with the UDD-AL model slightly outperforming high-T MD-AL models.

The situation is more interesting in C=O double bond scan (Supplementary Figure 2b). The model trained on 350K MD-AL data significantly deviates from the reference DFT curve beyond 1.36 Å separation with the overall RMSE of 3.9 kcal/mol. The 600K MD-AL model also exhibits poor performance with substantial underestimation of long stretch energies and the overall RMSE of 2.14 kcal/mol. Meanwhile, the model trained on low-T 350K UDD-AL data performs reasonably well over the entire bond length coordinate with the RMSE of only 0.92 kcal/mol. This observation becomes even more interesting when bearing in mind the fact that 350K UDD-Al and regular 600K MD-AL training sets result in nearly the same UMAP plots (Figures 2d and 2e in main text). Moreover, according to the histogram plot in Fig.1b (main text), the 600K MD-AL training set has a higher mean energy of 22.0 kcal/mol *vs.* 19.1 kcal/mol in 350K

UDD-Al training data. The 1000K MD-AL model achieves nearly the same overall accuracy with an RMSE of 0.90 kcal/mol. Therefore, the minimum sampling temperature required for accurate prediction of C=O stretch energies lies somewhere in the range 600-1000K for the regular MD-AL approach. At the same time, UDD-AL handles this task at a relatively low 350K.

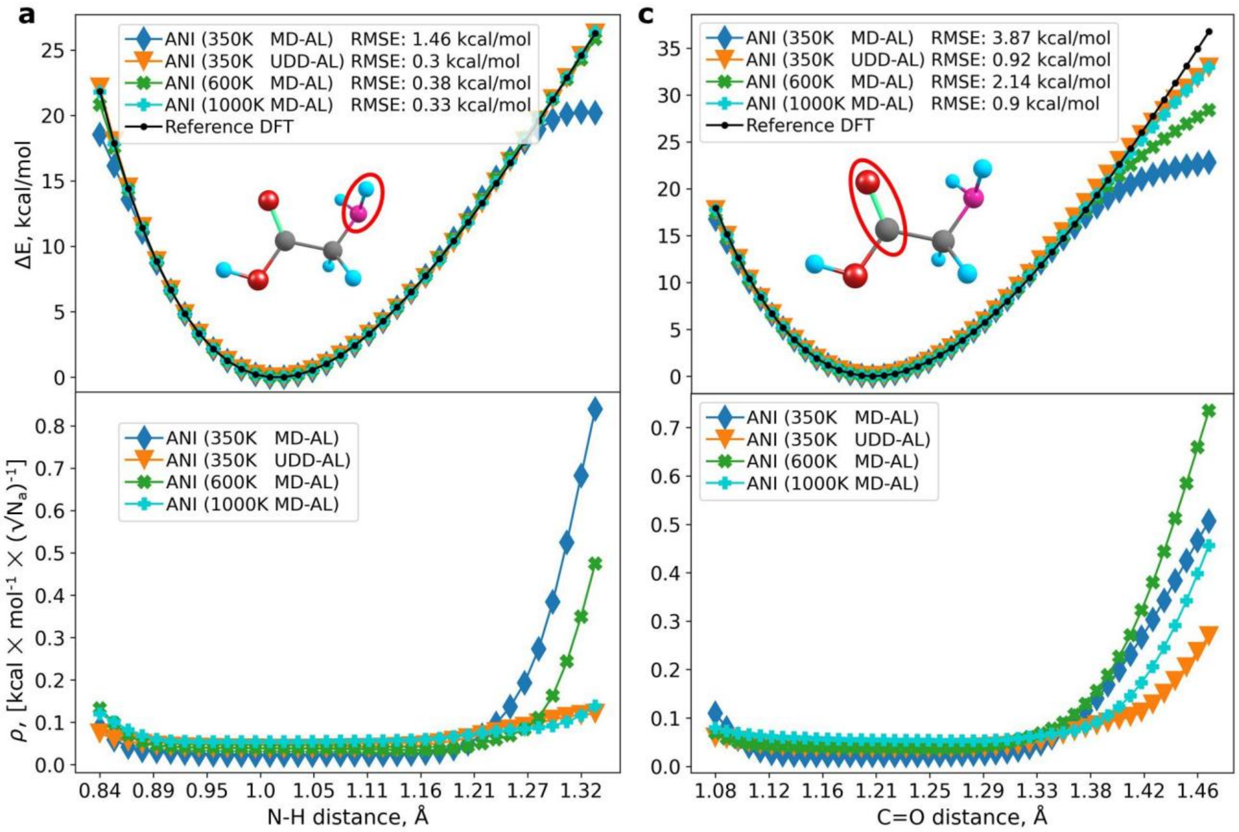

Supplementary Figure 2 | Models' performance on bond length scans. Each subplot shows a potential energy scan generated from reference DFT and models trained on 350K MD-AL (blue), 600K MD-AL (green), 1000K (cyan) MD-AL, and 350K UDD-AL (orange) data and also the corresponding ensemble uncertainty (bottom). Each line is averaged over three ensembles, each trained on data from an independent AL procedure. **a** Potential energy scan of N-H bond. **b** Potential energy scan of C=O bond. Atom colors: H – blue, C – grey, N – pink, O – red.

The rest of the bond length scans are provided in Supplementary Figures 3 and 4. The general trend is that UDD-AL model performs better than the rest of models (except for the C-C bond scan where UDD-AL model has RMSE of 0.43 kcal/mol against 0.39 for 1000K MD-AL)

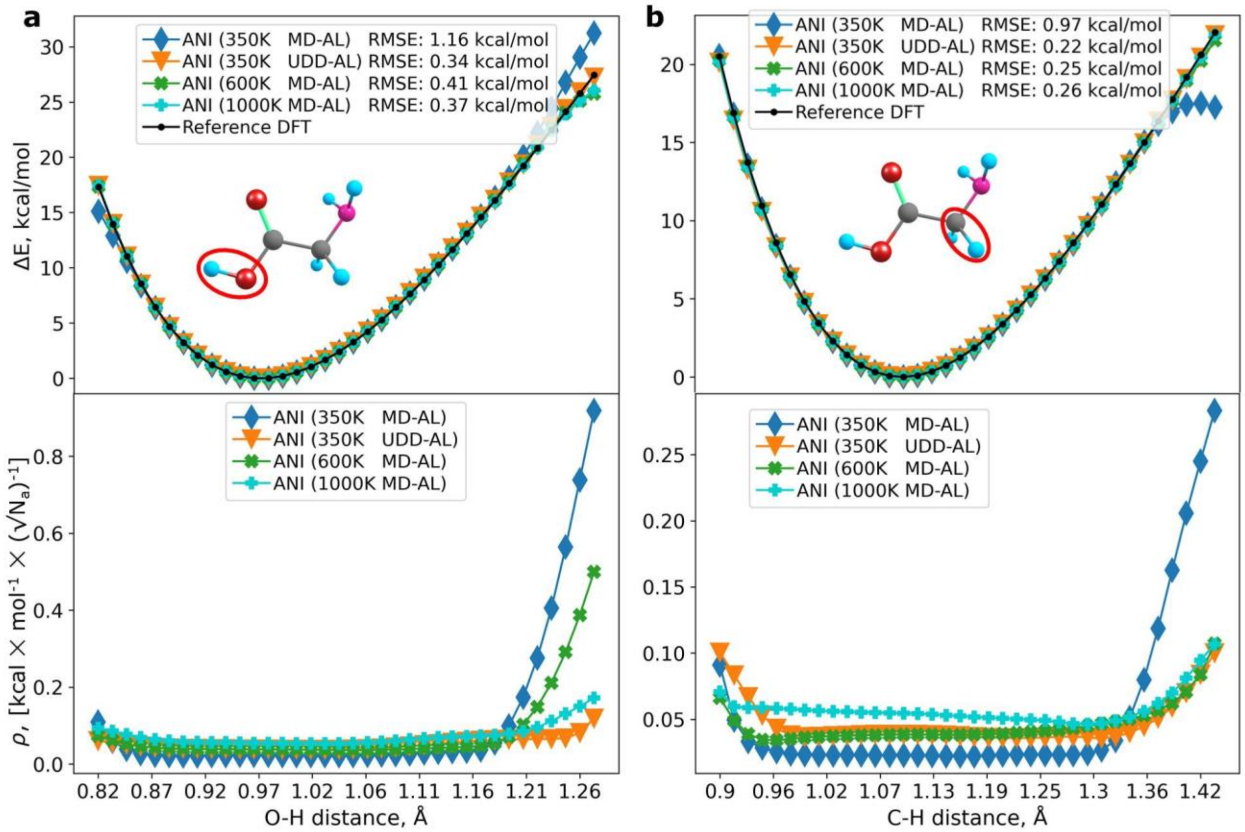

Supplementary Figure 3 | Models' performance on bond length scans involving H atom. Each subplot shows a potential energy scan generated from reference DFT and models trained on 350K MD-AL (blue), 600K MD-AL (green), 1000K (cyan) MD-AL, and 350K UDD-AL (orange) data and also the corresponding ensemble uncertainty (bottom). Each line is averaged over three ensembles, each trained on data from an independent AL procedure. **a** Potential energy scan of O-H bond length. **b** Potential energy scan of C-H bond length. Atom colors: H – blue, C – grey, N – pink, O – red.

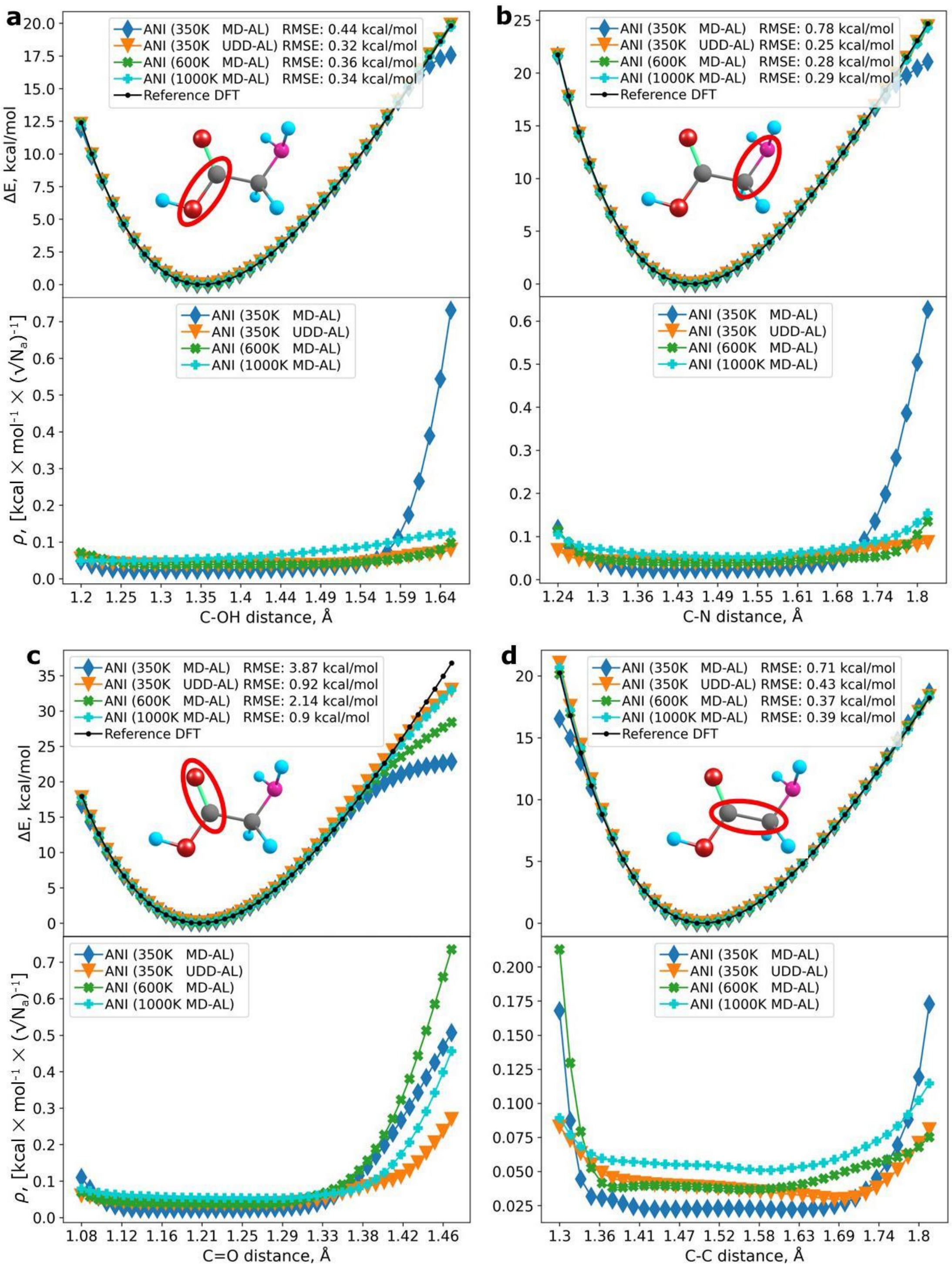

Supplementary Figure 4 | Models' performance on bond length scans not involving H atom. Each subplot shows a potential energy scan generated from reference DFT and models trained on 350K MD-AL (blue), 600K MD-AL (green), 1000K (cyan) MD-AL, and 350K UDD-AL (orange) data and also the corresponding ensemble uncertainty (bottom). Each line is averaged over three ensembles, each trained on data from an independent AL procedure. **a** Potential energy scan of C-OH bond length. **b** Potential energy scan of C-N bond length. **c** Potential energy scan of C-C bond length. **d** Potential energy scan of C=O bond length. Atom colors: H – blue, C – grey, N – pink, O – red.

Supplementary Section 3: Cross tests of UDD-AL and MD-AL models

Supplementary Table 2 summarizes RMSEs of the four models on the test sets accumulated by each AL sampler: 350K MD-AL, 350K UDD-AL, 600K MD-AL, and 1000K MD-AL. Diagonal values (italic font) represent models' performance on test sets that are generated using the same corresponding sampler. When considering the cross-tests without the diagonal elements, i.e. testing models on data that are not generated by the same corresponding sampler, the UDD-AL model outperforms all other models. Below we provide a detailed discussion on cross-tests of UDD-AL and MD-AL model pairs.

350K MD-AL vs 350K UDD-AL. The 350K MD-AL model, expectedly, has the lowest RMSE of 0.25 kcal/mol on the low-energy 350K MD-AL test set but exhibits the largest RMSE (4.78 kcal/mol) on the UDD-AL test set. Meanwhile, the UDD-AL model still has a relatively low RMSE of 0.34 kcal/mol on the 350K MD-AL test set and the lowest RMSE 0.65 kcal/mol on its own - 350K UDD-AL - test set.

600K MD-AL vs 350K UDD-AL. The UDD-AL model has the lowest error on both 600K MD-AL and 350K UDD-AL test sets. Although the difference is not that large for the 600K MD-AL test set (RMSE 0.57 vs 0.62 kcal/mol), the UDD-AL model performs much better than the 600K MD-AL model on the 350K UDD-AL test set (RMSE 0.65 vs 0.92 kcal/mol). These results are very unexpected when considering that the 600K MD-AL training set has an energy histogram and UMAP plot quite similar to the ones of a UDD-AL training set.

1000K MD-AL vs 350K UDD-AL. It turns out that the 1000K MD-AL test set is the most challenging one: all models exhibit the highest RMSEs here including the 1000K MD-AL model. Only the 1000K MD-AL model trained mostly on the corresponding high energy data achieves the RMSE of 0.87 kcal/mol. However, among the rest of the models, UDD-AL has much lower RMSE of 1.28 kcal/mol than 9.93 and 2.72 kcal/mol for 350K MD-AL and 600K MD-AL models, respectively.

Supplementary Table 2: Models' RMSEs on the test set comprising data from 350K MD-AL, 350K UDD-AL, 600K MD-AL, and 1000K MD-AL. Each of the four AL samplers was independently performed three times using the same initial training set: one AL data set was used for training and two others were used as a test set. Data from iterations 0-14 is not included since the bias is off, or the temperature is not increased at this stage. Thus, each AL run provides 1280-240=1040 data points. That is, each test set contains 2x1040=2080 glycine conformers. **Bold** values represent the best performing model on a test set that is not generated using the corresponding sampler. *Italic* values represent models' performance on a test set that is generated using the corresponding sampler.

Training data / Test data	350K MD-AL	350K UDD-AL	600K MD-AL	1000K MD-AL
350K MD-AL	*0.25*	**0.34**	0.41	0.37
350K UDD-AL	4.78	*0.65*	0.92	**0.73**
600K MD-AL	4.43	**0.57**	*0.62*	0.67
1000K MD-AL	9.93	**1.28**	2.72	*0.87*
